# 18p Deletion Syndrome With a 45, XY, t (14;18) (p11.1; p11.1), Karyotype

**DOI:** 10.7759/cureus.55539

**Published:** 2024-03-05

**Authors:** Baraah Ashgan, Abdulmoein Al-Agha, Yara Alhamdani, Mohamed Abdelmaksoud Shazly

**Affiliations:** 1 Pediatrics, Faculty of Medicine, King Abdulaziz University Hospital, Jeddah, SAU; 2 Pediatric Endocrinology, Faculty of Medicine, King Abdulaziz University Hospital, Jeddah, SAU

**Keywords:** short stature, dysmorphic feature, growth hormone deficiency, translocation, 18p deletion syndrome

## Abstract

Monosomy 18p deletion syndrome is a rare genetic disorder. We present an uncommon case of 18p deletion syndrome originating from a unique translocation between chromosomes 14 and 18 in an 11-year-old Saudi male, manifesting various clinical features. This case highlights the importance of understanding the genotype-phenotype correlations of 18p deletion syndrome to aid in the early recognition of the syndrome for its effective diagnosis and management.

## Introduction

Monosomy 18p (18p deletion syndrome), which was first identified by the French geneticist Jean de Grouchy in a patient in 1963, is characterized by the loss of genetic material from the short arm of chromosome 18 [1]. In the intricate tapestry of human genealogy, even minor alterations in the chromosomes can have profound implications, leading to remarkable clinical manifestations. The incidence of monosomy 18p is one in 50,000 newborn infants, with a male-to-female ratio of 2:3. Despite its rarity, over 150 patients with monosomy 18p have been documented globally with various features [2].

Monosomy 18p manifests various clinical features including growth retardation, intellectual disability as well as craniofacial deformities such as round face, wide mouth, microcephaly, and short neck [2]. To the best of our knowledge, this report presents the first case in Saudi Arabia of chromosome 18p deletion syndrome originating from a unique unbalanced translocation between the short arms of both chromosomes 14 and 18.

## Case presentation

**Figure 1:**
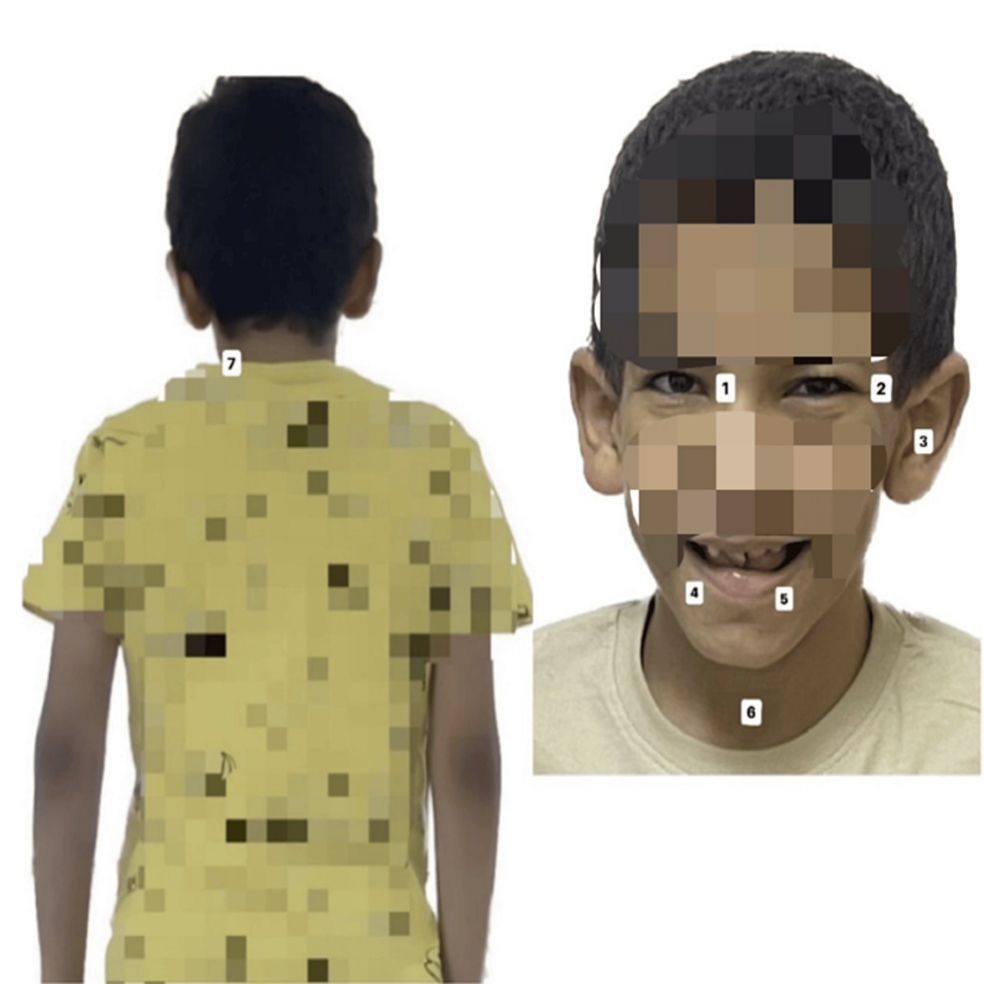
Dysmorphic features of the patient 1: strabismus, 2: ptosis, 3: large protruding ears, 4: dental abnormality, 5: corrected cleft palate, 6: short neck, 7: lower posterior hairline

The patient was an 11-year-old Saudi male who had been referred to the endocrine clinic at four years of age due to short stature; his height was 86.3 cm (-3.84 SD), and his weight was 14 kg (-1.32 SD); he also had facial dysmorphism. He had been born full-term, from a consanguineous marriage, with a birth weight of 3 kg and unremarkable perinatal, natal, or postnatal history, He had a positive history of delayed speech and language as his first word had been spoken at the age of three and a half years, and recurrent otitis media recurrent dental caries, as well as a past medical history of operated cleft palate. His systematic review and developmental milestones had been normal apart from mild intellectual disability, delayed speech, and dysarthria. Upon examination, the patient had multiple dysmorphic features (Figure 1). Other systemic examinations were unremarkable.

**Figure 2:**
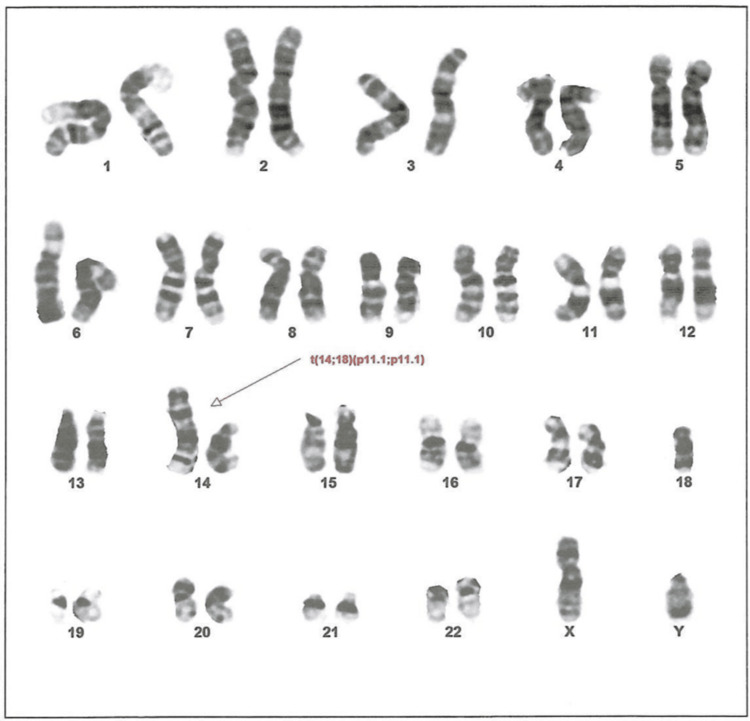
The karyotype test result of the patient The results show 45, XY, der(14;18) t(14;18)(p11.1;p11.1), which represents a male karyotype with a derivative chromosome resulting from a translocation between the short arm of chromosome 14 and the short arm of chromosome 18 at breakpoints (p11.1;p11.1), leading to monosomy 14p (chromosome 14 short arm deletion) and monosomy 18p (chromosome 18 short arm deletion).

**Figure 3:**
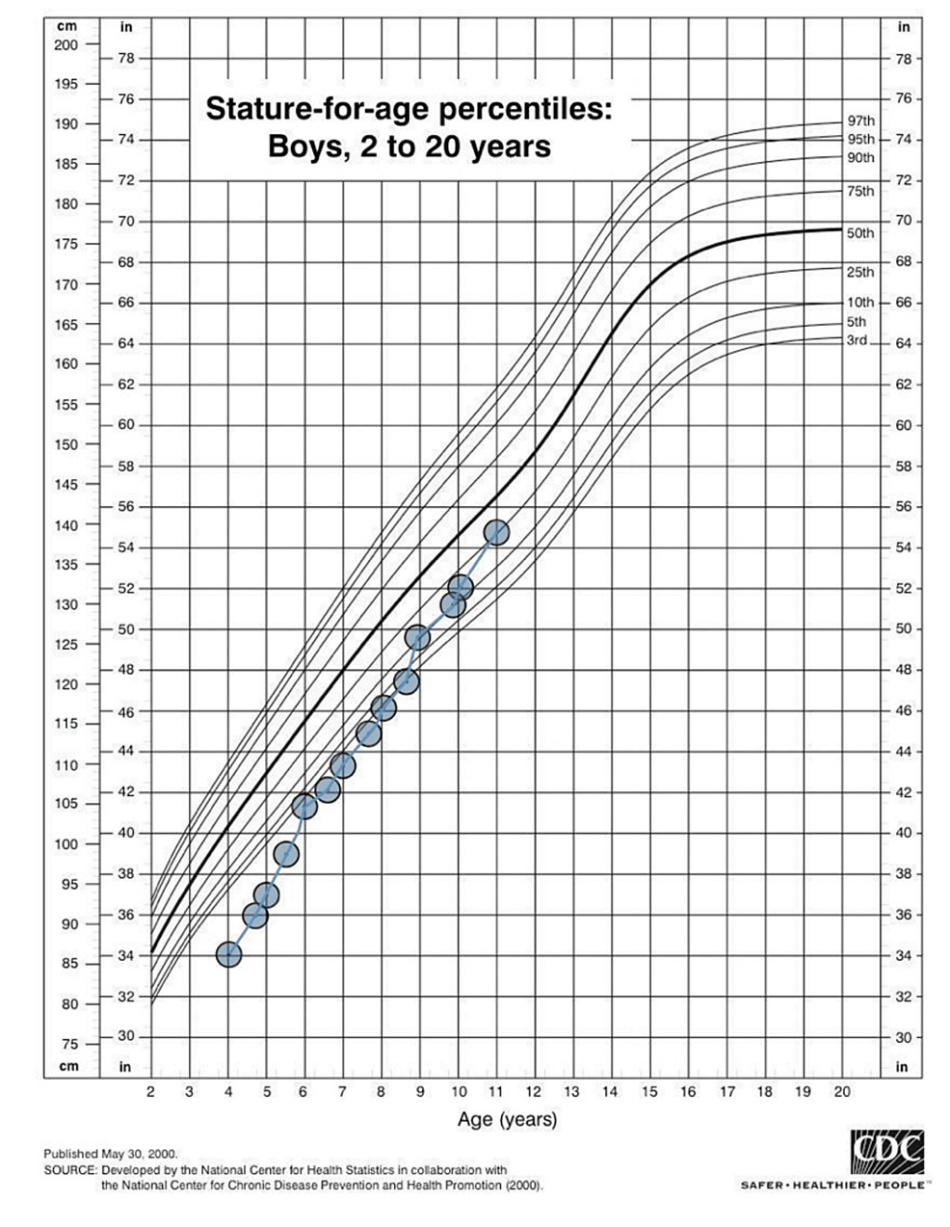
CDC height growth chart of the patient from 4 to 11 years of age Initially, the patient was -3.84 SD below the mean; after six years of growth hormone therapy, this improved to 0.65 SD below the mean CDC: Centers for Disease Control and Prevention

Multiple workups performed, including complete blood count, electrolytes, celiac serology, thyroid function test, luteinizing hormone, and follicular stimulating hormone, were within normal ranges. On the other hand, chromosomal analysis result was obtained as showed in (Figure 2). A growth hormone stimulation test confirmed a deficiency of growth hormone, and after obtaining consent from the family, growth hormone therapy was initiated at a dosage of 0.03 mg/kg administered subcutaneously once daily, which led to remarkable outcomes (Figure 3).

## Discussion

The human karyotype is composed of 46 chromosomes, arranged in 23 pairs, including 22 pairs of autosomes and one pair of sex chromosomes [3]. Chromosome 18 is notable for its relatively low number of protein-coding genes compared to other chromosomes. However, it contains a significant amount of conserved non-gene regions, which is not fully understood. However, some studies have suggested their importance in the genome [4]. Deletion on the short arm of chromosome 18 leads to a rare condition known as 18p deletion syndrome or monosomy 18. This syndrome usually appears to be caused by spontaneous errors very early in embryonic development [2]. In this proband, the karyotype test was abnormal, showing that he had 45, XY chromosomes with one derivative chromosome resulting from deletions in the short arms of chromosomes 18 and 14 originating from an unbalanced translocation between them.

This case report presents an unusual and rare condition involving combined structural chromosomal abnormalities. However, interestingly, the patient displayed symptoms consistent with 18p deletion syndrome despite concurrent chromosome 14 short-arm deletion; deletions of the short arms of any of the five acrocentric chromosomes including chromosome 14 do not cause significant phenotypic consequences since they possess similar sequences and hence the remaining short arms can compensate functionally [5]. Monosomy 18p is characterized by various clinical features, including intellectual disability, growth retardation, speech delay, behavioral abnormalities, and varying ranges and degrees of dental, ocular, and craniofacial abnormalities, such as webbed or short neck with low posterior hairline, and large ears [2].

Speech delay is one of the common manifestations of this condition, which might be due to a cleft palate or lip. However, several case reports of monosomy 18p have documented speech delays without a history of cleft palate or lip [6,7]. On the other hand, in contrast to previous case reports documenting numerous other anomalies such as abnormal genitalia and heart defects, our patient did not have any previously mentioned anomalies [8,9]. A 2006 study involving seven individuals with 18p deletion syndrome proposed that a crucial area, particularly between 18p11.1 and 18p11.21, might be linked to the onset of intellectual disability. Our patient, who had a mild intellectual disability, was found to have a deletion at 18p11.1 through karyotype analysis. This finding endorses the hypothesis presented in the earlier study [2].

Previous reports have suggested a link between monosomy 18p and growth hormone deficiency. A recent review of individuals with 18p deletion syndrome reported that 23% of them had a deficiency in growth hormone and these individuals responded positively to growth hormone therapy, highlighting the significance of early treatment [10]. Our patient was diagnosed with a deficiency of growth hormone and showed a remarkable response to growth hormone replacement therapy, which aligns with the findings of the first documented case of a patient with 18p deletion syndrome, reported back in 1992, who also benefited from growth hormone therapy [11].

## Conclusions

It is important to report such cases exhibiting unique genetic variations as it would contribute to understanding the genotype-phenotype correlations, thereby enhancing healthcare-related knowledge of the specific features associated with 18p deletion syndrome; it would also aid in differentiating the condition from other syndromes sharing similar clinical features. We recommend reporting more cases of 18p deletion syndrome and adopting a multidisciplinary approach to provide optimal management depending on the various clinical manifestations of the condition.
